# *Macaca mulatta* is a good model for human mandibular fixation research

**DOI:** 10.1098/rsos.220438

**Published:** 2022-11-16

**Authors:** Pranav N. Haravu, Hyab Mehari Abraha, Michelle Shang, Jose Iriarte-Diaz, Andrea B. Taylor, Russell R. Reid, Callum F. Ross, Olga Panagiotopoulou

**Affiliations:** ^1^ Department of Surgery, Section of Plastic Surgery, The University of Chicago Medical Centre, Chicago, IL, USA; ^2^ Monash Biomedicine Discovery Institute, Department of Anatomy and Developmental Biology, Monash University, Victoria, Australia; ^3^ Department of Biology, The University of the South, Sewanee, TN, USA; ^4^ Department of Basic Science, Touro University, Vallejo, CA, USA; ^5^ Department of Organismal Biology and Anatomy, University of Chicago, Chicago, IL, USA

**Keywords:** mandible angle fracture, Champy fixation, biplanar fixation, chewing, rhesus macaque, finite-element model

## Abstract

Biomechanical and clinical studies have yet to converge on the optimal fixation technique for angle fractures, one of the most common and controversial fractures in terms of fixation approach. Prior pre-clinical studies have used a variety of animal models and shown abnormal strain environments exacerbated by less rigid (single-plate) Champy fixation and chewing on the side opposite the fracture (contralateral chewing). However, morphological differences between species warrant further investigation to ensure that these findings are translational. Here we present the first study to use realistically loaded finite-element models to compare the biomechanical behaviour of human and macaque mandibles pre- and post-fracture and fixation. Our results reveal only small differences in deformation and strain regimes between human and macaque mandibles. In the human model, more rigid biplanar fixation better approximated physiologically healthy global bone strains and moments around the mandible, and also resulted in less interfragmentary strain than less rigid Champy fixation. Contralateral chewing exacerbated deviations in strain, moments and interfragmentary strain, especially under Champy fixation. Our pre- and post-fracture fixation findings are congruent with those from macaques, confirming that rhesus macaques are excellent animal models for biomedical research into mandibular fixation. Furthermore, these findings strengthen the case for rigid biplanar fixation over less rigid one-plate fixation in the treatment of isolated mandibular angle fractures.

## 1. Introduction

Choosing the best technique to fix a fractured mandible is an important topic in oral and maxillofacial surgery. Despite years of research, there is ongoing debate among surgeons on the best treatment method for angle fractures, one of the most common mandible fracture types in adults [1]. Treatment of angle fractures involves the alignment and reduction of the fracture, accompanied by fixation using titanium miniplates placed surgically across the fracture and secured using monocortical screws. The number of plates/screws and their orientation depends on the fracture site. In the case of an angle fracture, fixation can be less rigid or more rigid.

Less rigid fixation allows some motion at the fracture line and typically involves the transoral placement of one miniplate (Champy method, figure 1.3) at the external oblique ridge of the mandible [2–5]. The alternate more rigid treatment augments the Champy method with an additional plate placed trans-buccally on the lateral inferior border of the mandible (biplanar fixation, figure 1.3) [5–8]. While biplanar fixation is theorized to be more stable, it is often less preferred surgically because it is more invasive and requires an extraoral scar and reflection or splitting of the masseter muscle [9–11]. The Champy method avoids muscular disruption and tends to be operatively easier [12], making it the most popular fixation technique to date [5,10,13,14].

**Figure 1.**
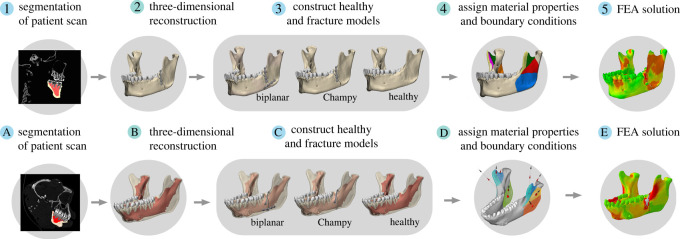
Flow chart of finite-element analysis (FEA) for human models (1–5) and previously published macaque models (A–E). Patient specific computed tomography scans were processed to create three-dimensional (3D) models of the healthy controls and the fracture fixation angle treatments. All models were assigned analogous tissue material properties and boundary conditions to simulate unilateral post-canine chewing and solved using Abaqus static implicit solvers. The same process was used for macaque models. Both human and macaque models had teeth, trabecular bone and cortical bone segmented. Graphical representation of human models in 2 and 3 shows the cortical bone, while the graphical representation of the macaque models in B and C shows the underlying trabecular bone.

**Figure 2.**
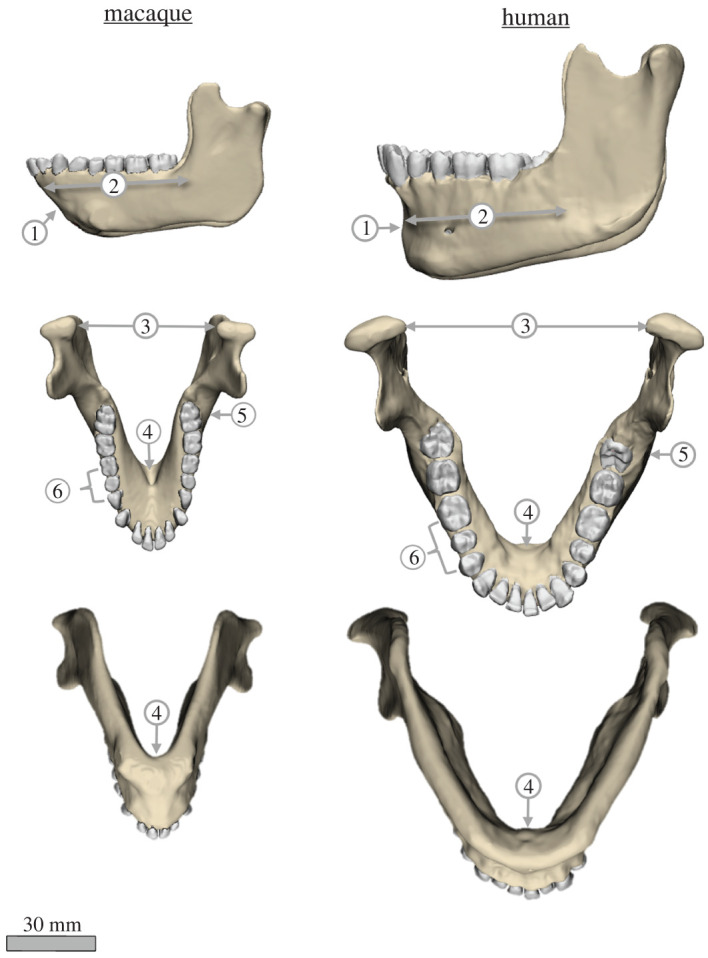
Comparison of mandible morphology between humans and macaques. Lateral (top row), superior (middle row) and inferior (lower row) views of the rhesus macaque and human (*Homo*) mandibles, figures to scale. Important differences in mandible morphology include: (1) more vertical surface of anterior symphyseal region and presence of a chin in *Homo*; (2) relatively shorter mandibular corpus and relatively greater AP width of the ramus in *Homo*; (3) relatively greater inter-condylar distance in *Homo*; (4) reduced inferior transverse torus and relatively smaller AP dimension of symphyseal region in *Homo*; (5) more posteriorly located M_3_ in *Homo*, (6) positioning the distal end of M_3_ medial/lingual to the anterior edge of the ramus.

However, in a recent study in rhesus macaques, we showed that the less rigid and most commonly used repair technique, the Champy method, results in a biomechanically unfavourable environment in the mandible. This includes greater interfragmentary strain at the fracture plane, higher bone strain magnitudes in the bone-implant interface, and global bone strain regimes that deviate more from the healthy control than the biplanar (more rigid) fixation [15]. These unfavourable biomechanical environments are exacerbated during contralateral chews (chewing on the opposite side to the fracture). This partly occurs because the mandible undergoes lateral transverse bending during unilateral post-canine chewing [16,17]. Champy fixation provides minimal resistance against lateral transverse bending, and thus is associated with greater widening of the medial fracture plane during contralateral chews than biplanar fixation [15]. In addition, under contralateral chewing, Champy fixation reverses the twisting moments acting about the anterior–posterior (AP) axis of the fracture-fixed mandible, such that anterior to the fixation construct the corpus is subjected to negative twisting moments (eversion of the tooth row and inversion of the inferior border). This results in opposite twisting moments occurring on either side of the fracture plane, increasing the strains in the plate construct and in the bone-implant interface [15]. Together, these findings suggest that chewing contralateral to the fracture side following Champy fixation probably results in biomechanical conditions (high strain, high interfragmentary instability) that inhibit bone healing. However, there are notable morphological differences between macaques and humans (figure 2). These differences make it imperative to compare the biomechanical performance of macaque and human mandibles in both healthy and post-fracture fixation states before macaque results can be used to guide clinical decision-making.

Previous studies on mandible biomechanics in healthy rhesus macaques during unilateral post-canine chewing have shown that the overall deformation pattern of the macaque mandible is remarkably similar to that of humans [17–19]. During unilateral post-canine chewing, both the human [19,20] and macaque [16,17,21] corpora are twisted about their long axis such that there is inversion of the balancing side toothrow and eversion of the working side toothrow. Similarly in both humans and macaques the balancing side corpus uniformly experiences negative sagittal bending [17,22], while working side corpus sagittal bending direction depends on the location of the bite point (negative posterior and positive anterior to bite point) [17,22]. Lastly, in both species, the symphyseal region predominately undergoes lateral transverse bending and negative mediolateral twisting [17,22].

Despite these similarities in deformation regimes between humans and macaques, there are noteworthy morphological differences between the mandibles of the two species (figure 2).

Compared with *Macaca*, *Homo* has a more distally positioned toothrow and relatively shorter mandibular corpus, decreasing the load arm of the bite force, and increasing relative bite force for the same input muscle force. However, humans also have relatively smaller chewing muscles than macaques, perhaps offsetting the expected increase in bite force [23,24].

*Homo* also has more divergent rami than cercopithecines (a subfamily of Old World monkeys), associated with a relatively greater inter-condylar distance. The greater divergence of the rami in the human mandible is expected to increase the ML component of the medial pterygoid muscle and decrease the mediolateral (ML) component of the superficial masseter [25]. Consequently, resultant AP twisting moments acting to invert the tooth row are likely to be higher in humans than in macaques. Lastly, the increase in rami divergence also results in a greater mediolateral inclination of the toothrow in humans compared with macaques. As a result, bite points posterior to P_4_ (i.e. the posterior premolar) are medial to the long axis of the mandible in humans, but over that axis in macaques. Thus, twisting moments acting about the AP axis at these bite points are likely to be higher in humans than in macaques.

Symphyseal morphology is also markedly different between adult humans and macaques. The macaque lingual symphysis is dominated by the superior and inferior transverse tori and is obliquely inclined [26], whereas the inferior transverse torus is absent in humans and the symphysis is more vertically oriented [27,28]. As a result, the labiolingual thickness of the symphyseal region is greater in macaques than in humans. In addition, the dental alveolar and basal mandibular arches [29] are less acute in humans than in macaques [21]. Similar differences are seen in the inner mandibular arch, consisting of the superior transverse torus, medial prominence and alveolar prominence. These differences have implications for the distribution of strains along the lingual and labial surfaces of the mandible. If the mandible is treated mechanically as a curved beam, the increased symphyseal curvature in the macaque jaw should result in increased lingual tensile strains (concave surface) and decreased labial compressive strains (convex surface) relative to the human, when the jaw undergoes lateral transverse bending [16,30]. However, the increased labiolingual thickness of macaques strengthens the mandible against the dominant loading regimes in the symphysis during the power stroke of mastication—lateral transverse bending and dorsoventral shear [16,31,32]. Thus, one might predict that any differences in labiolingual strains introduced by the more acute mandibular arch in macaques are ameliorated by the relative increase in labiolingual thickness [30,31]. As a result, differences in symphyseal morphology between humans and macaques may not significantly alter deformation and strain regimes.

The aim of this study is to compare the deformation and strain regimes of the macaque and human mandible in healthy (non-fractured) and post-fracture fixation conditions to determine whether rhesus macaques are suitable animal models for oral and maxillofacial research. For the purpose of this comparison, we are excluding the potential impact of morphological variation due to ontogeny [33–35], as our objective is to determine whether an adult macaque can serve as an appropriate animal model for testing interventions in adult human patients. We hypothesize that deformation and strain regimes will be similar in adult human and macaque mandibles, in line with the previous findings of Panagiotopoulou *et al*. [17], Korioth *et al*. [18]; Korioth & Hannam [19]; Korioth & Versluis [22]. In addition, given the proposed similarities in deformation regimes between the two species under healthy conditions, we propose that fracture and fixation conditions will have the same effect in both models. As was observed in macaques [15], we expect that Champy fixation in humans will create larger effects on twisting moments acting about the jaw, increased interfragmentary strain, and global principal strain regimes that differ more from the healthy condition.

## 2. Methods

### 2.1. Overview

A detailed description of our methods is provided below; they consisted of the following steps. Finite-element models were built from the post-surgical fixation CT scans of an adult male patient who had an angle fracture of the mandible repaired with Champy fixation. *In silico* processing was used to simulate the addition of a second plate to create a model representing biplanar fixation, as well as a third model in which the plate was removed, and the fracture was spanned with bone to represent the healthy state. Each of these models was loaded with muscle forces and boundary conditions to represent ipsilateral and contralateral chewing, and the resulting strain, moments and displacement were analysed. The results of this analysis were compared with previously validated macaque models.

### 2.2. Macaque models

The healthy and post-fracture fixation macaque models were constructed as part of previous publications [15,17,36,37], where details for model creation and solution can be found. In short, the macaque models were built using the same methodology as described below for the human models, with *in silico* editing to create biplanar and Champy fixation models in addition to the healthy model. Material properties, muscle activation patterns, and boundary conditions were the same for both macaque and human models.

### 2.3. Human models—Champy fixation

We created a finite-element (FE) model of the human mandible from an adult male patient treated surgically for a left side angle and symphyseal fracture at the University of Chicago in 2016. The patient underwent open reduction and internal fixation using the Champy technique performed by one of the co-authors (R.R.R.), specifically with a 0.6 mm thick four-hole miniplate and non-locking screws (Zimmer Biomet, Inc.). We captured the three-dimensional geometry of the mandible and cranium using post-surgical diagnostic CT scans obtained from the University of Chicago Trauma Centre under IRB protocol IRB17–1057. Non-contrast maxillofacial CT examinations were performed on a Phillips Brilliance multidetector-row CT scanner (16-, 64- or 256-slice; Philips Medical Systems, Best, The Netherlands). The detector collimation was 0.625 mm; scan thickness was 0.8 mm, with an increment of 0.4 to 0.5 mm (figure 1.1). In Mimics v. 21.0, we isolated the mandible and cranium and separated the mandible tissues of interest to create three-dimensional (3D) surfaces of the cortical bone, trabecular tissue (modelled as solid structure), teeth, Champy miniplate and screws (figure 1.2). The periodontal ligament (PDL) was excluded as the resolution of the post-surgical diagnostic scans (0.4 mm × 0.4 mm × 0.8 mm) was not sufficient to accurately reconstruct the periodontal tissue (0.05 and 0.5 mm size—Nikolaus *et al*. [38]). In addition, we previously showed that the PDL has a negligible effect on global strain regimes in the healthy macaque mandible [39]. In 3-Matic v. 15.0, the three-dimensional surface files of the implants and mandibular tissues (cortical bone, trabecular tissue, teeth) were compiled into a non-manifold assembly. A concurrent symphyseal fracture was repaired *in silico* using automatic and manual mesh techniques (figure 1.3). The assembly was then converted into volumetric mesh files of 1 mm linear tetrahedral elements and exported to Abaqus CAE Simulia software (Dassault Systémes, Vélicy-Villacoublay, France) for modelling (electronic supplementary material, table S1). In all models, isotropic and homogeneous material properties were assigned to the cortical bone (*E* = 17 GPa; *v* = 0.3), teeth (*E* = 24.5 GPa; *v* = 0.3), trabecular tissue (*E* = 10 GPa; *v* = 0.3) and implant materials (*E* = 105 GPa; *v* = 0.36).

### 2.4. Human models—healthy and biplanar variants

We created a healthy mandible (control) by removing the miniplate from the mandible of the patient and reconstructing the fracture line in 3-Matic v. 15.0 with a combination of automatic and manual mesh techniques (figure 1.3). To test the effect of different angle fracture techniques compared with our healthy control, we created a biplanar variant of the Champy fixation by adding a titanium miniplate across the fracture plane at the inferior lateral border of the mandible. The inferior miniplate was 26.1 mm × 2.5 mm × 1 mm and the screws were modelled as locking screws. All model components (cortical bone, trabecular bone tissue, teeth, miniplates and screws) were collated into a non-manifold assembly (figure 1.3 and 1.4), converted to volumetric files, and exported to Abaqus Simulia CAE 2019 for processing.

### 2.5. Human models—boundary conditions and muscle loads

All adjacent biological surfaces (cortical bone–teeth, trabecular bone–teeth, cortical bone–trabecular bone) were bound together with tie constraints. Due to modelling limitations the interfaces between the screws and the miniplate were also modelled as tie constraints. The use of tie constraints approximates the screw–miniplate interface as a locking screw as opposed to the non-locking screws used in the surgical fixation, but recent studies have shown no significant difference in horizontal stiffness, vertical stiffness, or rupture strength between the two screw types [40]. The adjacent surfaces of the fracture plane and the interactions between the miniplate and bone were treated as ‘hard’ contacts and assigned the same penalty coefficient of static friction as the macaque models (0.3) [41,42].

To simulate unilateral post-canine chewing, we assigned to the human models the same constraints that were used in the macaque models [17,37]. The chewing side condyle and bite points (both premolars and first molar) were constrained against translation in all directions. To investigate the impact of constraints on model behaviour, we also created versions of the models in which the bite points were constrained only in the vertical direction, and the resulting strain regimes are presented in electronic supplementary material, figure, S1*A*,*B*,*C*. Given the overall similarities between the two bite-point constraints we chose to focus on presenting the constraints that have been validated in our prior macaque models. The non-chewing side condyle was constrained against translation in the anterior–posterior and superior–inferior directions [17,37]. To load the models, we selected nodes on the mandible that represent the origin and insertion of each major jaw-closing muscle (anterior and posterior temporalis; deep and superficial masseters; medial pterygoids) based on literature (figure 1.4) and applied the respective muscle force vector across all surface nodes (table 1). The force vectors' direction was calculated by measuring the angle of the vector from the centroid of the mandibular insertion to the centroid of the cranial origin [17,37]. The magnitude of each force vector was calculated by scaling the maximum force of the muscle by the proportion of activation used during nut chewing in macaques. The maximum force was calculated by multiplying each muscle's estimated physiological cross-sectional area (PCSA) by the specific tension of muscle (30 N cm^−^^2^), which is a conservative estimate of muscle stress [43]. Proportions of maximal activation were based on published electromyography (EMG) data recorded from the same rhesus macaque during unilateral chewing and represent peak muscle activation at maximum strain [17,37]. This method of calculating muscle forces ensures that differences in deformation and strain between macaques and humans are due to the differences in mandibular shape and size rather than variability in applied muscle activation patterns [44]. All models were solved using the Abaqus v. 2019 direct implicit static solver and the average solution time (eight processors and four tokens) was approximately 10 min for each model (figure 1.5).

**Table 1.** Muscle force vector (*N*) assigned to all human FE models.

	muscle	SI (X)	AP (Y)	ML (Z)
left side (ipsilateral) chew	anterior temporalis L	58.4	3.8	0.7
	anterior temporalis R	80.4	6.9	−2.4
	posterior temporalis L	23.3	−16.1	5.4
	posterior temporalis R	53.1	−38.0	−15.6
	deep masseter L	8.2	0.3	3.0
	deep masseter R	40.1	9.0	−17.6
	superficial masseter L	107.7	23.8	22.5
	superficial masseter R	53.2	16.8	−13.9
	medial pterygoid L	58.1	28.0	−43.2
	medial pterygoid R	21.9	11.6	13.6
right side (contralateral) chew	anterior temporalis L	80.5	5.3	0.9
	anterior temporalis R	58.3	5.0	−1.7
	posterior temporalis L	54.2	−37.5	12.5
	posterior temporalis R	22.8	−16.3	−6.7
	deep masseter L	42.0	1.5	15.2
	deep masseter R	7.9	1.8	−3.5
	superficial masseter L	55.0	12.2	11.5
	superficial masseter R	104.2	32.9	−27.2
	medial pterygoid L	21.1	10.2	−15.7
	medial pterygoid R	60.2	31.8	37.3

Note: Coordinate system in our model has the origin in the midpoint between the condyles, *x*-axis is superior–inferior (SI), *y*-axis is anterior–posterior (AP), and *z*-axis is mediolateral (ML). L = left, R = right.

### 2.6. Data analysis

All macaque data have been previously published [15,17,36,37] and are presented here exclusively for ease of comparison with human models.

All plots of principal, normal and shear strains mapped onto the surface of the FE human models were exported from Abaqus v. 2019. All moments were calculated using the Abaqus Free Body Toolset (FBT). Abaqus FBT calculates the components of moments acting anterior to each coronal cross-section and to the left of each sagittal section by integrating the internal forces in all elements in each section. Coronal sections were used to calculate moments in each hemimandible, and sagittal sections were used to calculate moments in the symphysis. Thirty equidistant slices were taken of each hemimandible/section, and specific sections were selected from the set based on anatomically homogeneous locations between species (electronic supplementary material, figure S2).

Bone-implant interface strains were defined as cortical strains on the buccal surface of the mandible, proximal to the implant, with the region defined as the bounding box depicted in the corresponding bone implant figure. Specifically, coordinates and strain values for cortical surface nodes were exported from Abaqus CAE v. 2019 and processed in Python 3.9.6 with custom-written code. Nodes on interface surfaces (cortical bone and screws, cortical bone and fixation plate) were not included in the analysis. Nodes were selected based on their *x-*, *y-* and *z*-coordinates such that the maximum *x*-value was the superior edge of the oblique fixation plate, the maximum *y*-value was at the midpoint of M_2_, and the minimum *z*-value was the inferior border on the lingual surface of the mandible. The median principal strains were calculated for the selected nodes. In addition, selected cortical nodes were categorized by principal strain value to generate histograms representative of strain distribution.

Fracture plane analysis, to measure interfragmentary strain (IFS), provided a numerical and graphical representation of the change in width of the fracture plane before and after loading. Nodal coordinates from the fracture plane were exported from Abaqus CAE v. 2019 and processed in Matlab R2020a with custom-written code. The calculation first involved measuring the distance between two nearest nodes in the undeformed and deformed states. Interfragmentary strain was then defined as (deformed distance – undeformed distance)/undeformed distance. In the human models, this included 1120 nodes and 999 nodes for the biplanar and Champy fixations, respectively. Compressive and tensile regions were defined as node pairs that had negative interfragmentary strain (negative change in distance) and positive interfragmentary strain (positive change in distance), respectively.

Difference in principal strains between fixation and healthy models provided element-level graphical representation of differences in principal strains between models. Nodal strains were exported from the entire cortical bone surface of all models using Abaqus CAE v. 2019. Using custom-written Matlab R2020a code, the difference in elemental principal strains between healthy and fixation models was computed and then displayed on the cortical bone surface.

All anatomical terminology (electronic supplementary material, figure S3) is consistent with nomenclature used in the clinical literature (e.g. [12]) and in similar comparative studies of chimpanzee and macaque mandibles [17,44].

## 3. Results

### 3.1. Healthy models

#### 3.1.1. Muscle forces

The mandible of the macaque model is 76 mm long and the human is 113.5 mm, so if muscle forces were scaled isometrically, *Homo* muscle forces should be 2.25× those of *Macaca*. However, human muscle forces (PCSA data from [45,46]) range from 0.9× (medial pterygoid) to 2.5× (posterior temporalis) those of the macaque [17]. Medial pterygoid, superficial masseter (1.6×), anterior temporalis (2.0×) and deep masseter (2.1×) are all smaller than expected on the basis of isometry. The posterior temporalis is the only muscle that is relatively larger in the human model than in the macaque. All else being equal, relatively small jaw muscles in *Homo* are expected to be associated with relatively lower torques and strain magnitudes compared with *Macaca*.

Table 2 compares the unit vectors of the jaw muscles in *Macaca* and *Homo*. All muscles except the medial pterygoid are more vertically oriented (greater SI and lower AP components) in *Homo* than in *Macaca*. The superficial and deep masseters are also less ML oriented in *Homo* than in *Macaca.* The medial pterygoids of *Homo* are more ML oriented and less vertically oriented (both SI and AP) than in *Macaca*. During right-sided chew the bite force in the human models was 204.7 N, and during left-sided chew it was 197.7 N.

**Table 2.** Unit vector comparisons between *Homo* and *Macaca*. SI, superior–inferior; AP, anterior–posterior; ML, mediolateral.

	muscle direction (unit vector)								
	*Homo*			*Macaca*			*Homo − Macaca*		
	SI (X)	AP (Y)	ML(Z)	SI (X)	AP (Y)	ML (Z)	SI (X)	AP (Y)	ML (Z)
anterior temporalis L	1.00	0.07	0.01	0.96	−0.28	0.09	0.04	0.35	−0.08
anterior temporalis R	1.00	0.09	−0.03	0.97	−0.22	0.03	0.03	0.31	−0.06
posterior temporalis L	0.81	−0.56	0.19	0.65	−0.74	0.18	0.16	0.18	0.01
posterior temporalis R	0.79	−0.57	−0.23	0.66	−0.75	−0.07	0.13	0.18	−0.16
deep masseter L	0.94	0.03	0.34	0.72	−0.14	0.68	0.22	0.17	−0.34
deep masseter R	0.90	0.20	−0.39	0.75	−0.04	−0.66	0.15	0.24	0.27
superficial masseter L	0.96	0.21	0.20	0.79	0.49	0.36	0.17	−0.28	−0.16
superficial masseter R	0.93	0.29	−0.24	0.85	0.31	−0.43	0.08	−0.02	0.19
medial pterygoid L	0.75	0.36	−0.56	0.90	0.19	−0.38	−0.15	0.17	−0.18
medial pterygoid R	0.78	0.41	0.48	0.89	0.17	0.43	−0.11	0.24	0.05

### 3.2. Moments and deformation

**Figure 3.**
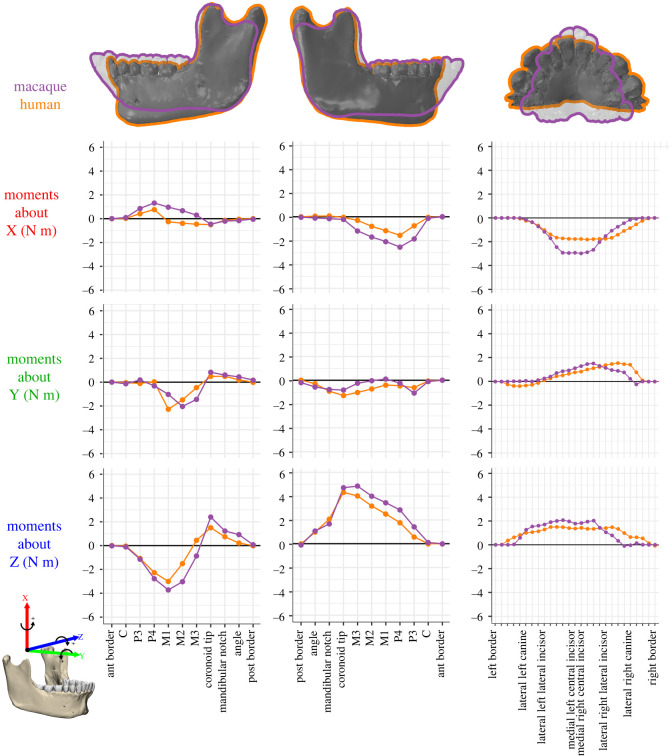
Moments (N m) acting anterior to coronal sections (for left and right hemi-mandible) and to the left of sagittal sections (for the symphysis) at landmark locations through the human (orange) and macaque (purple) mandible. Working side is the left and balancing side is the right. *Macaca* moments are scaled isometrically to the mandible length of the *Homo* model (length^3^). Note: macaque moment data were generated from previously published FE models [17] using the same moments analysis protocol applied to the human models.

Moments acting on human and macaque mandibles during a left-sided chew are compared in figure 3. To remove the effects of size, the moments for *Macaca* are scaled isometrically to the mandible length of *Homo*. The more vertically oriented muscles of *Homo* are associated with lower SI moments about vertical (X) axes than in the isometrically scaled macaque, but not with greater ML moments about transverse (Z) axes. This is because, even though most human jaw muscles are more vertically oriented than those of macaques, they are also relatively smaller. The only moments that are larger in *Homo* than in the scaled *Macaca* are AP twisting moments acting on the balancing side mandible. This is likely to be an effect of the more divergent rami in *Homo*.

In humans (orange on the graph), the working side SI (transverse bending) moments have a positive peak at P_4_ and are negative between M_1_ and the coronoid tip (figure 3). This indicates that the working side corpus experiences lateral transverse bending anterior to the bite point but medial transverse bending posterior to M_1_. Balancing side SI moments are negative from the M_3_ through to P_3_, with a minimum at P_4_ (figure 3), indicating lateral transverse bending. The working side AP (twisting) moments are negative between M_1_ and the M_3_, with small positive AP moments beginning at the coronoid tip and continuing to the posterior border of the ramus. From these twisting moments we infer that the working side predominately undergoes negative anterior–posterior twisting, such that the base is inverted while the toothrow is everted. Balancing side AP moments are negative from the posterior border of the ramus to the canines, with local minima at the coronoid tip and P_3_, indicating that the balancing side mandible also undergoes negative AP twisting, with basal eversion and toothrow inversion. Working side ML (sagittal bending) moments are negative between the canines and M_2_ and positive between M_3_ and the mandibular angle, indicating positive sagittal bending anterior to the M_3_ and negative sagittal bending posterior to the M_3_. Balancing side ML moments are positive across the ramus and corpus (figure 3)—reflecting negative sagittal bending. In the symphysis, negative SI (transverse bending) moments in humans are lower than in macaques and are negative from the left canine past the right canine. Positive AP moments at the human symphysis are similar in magnitude to the macaque, but peak more laterally, between the right lateral incisor and canine. ML moments at the symphysis are similar in magnitude and distribution between human and macaque models (figure 3).

The moments acting on a section anterior to the biting side M_2_ of the non-fractured mandible in *Macaca* and *Homo* are given in table 3. At this section, the human mandible experiences 2.47× greater torques about an AP axis (My) than macaques, much less than predicted by isometry (3.38). This is primarily due to the very low My torque generated by the bite force, a value of −0.84 N m, which is only 1.1× the macaque value. The low My torque is due to the positioning of the bite points being more directly over the mandible in humans than in macaques. The (right) balancing side anterior temporalis is also slightly negatively allometric; all other torques in humans are isometric or positively allometric. This suggests that the primary determinant of differences in My torques between macaques and humans is the position of the toothrow relative to the underlying corpus.

**Table 3.** Twisting moments (N m) acting about an AP (*y*) axis anterior to a coronal section through the working side (left) *M*_2_. Rt = right; Lt = left; Fx = forces in SI direction (vertical, positive is up); Fz = forces acting in mediolateral (ML) direction (positive is to animal left); dx = superior–inferior (SI) distance between section centroid and muscle attachment or reaction force centroid; dz = ML distance between section centroid and muscle attachment or reaction force centroid. Moments acting on the mandible behind the section of interest (working side M_1_) are 0, and are excluded. Fz is 0 at the balancing side condyle because the condyle is unconstrained in ML. For ratios: isometry of moments for a mandible 1.5× the size of *Macaca* is 3.38.

	macaque			human			human/macaque ratios		
	Fx × dz	Fz × dx	My torque	Fx × dz	Fz × dx	My torque	Fx × dz	Fz × dx	My torque
Rt anterior temporalis	−1.59	0.03	−1.62	−5.31	−0.09	−5.22	3.34	−2.95	3.22
Rt posterior temporalis	−0.91	−0.07	−0.84	−3.60	−0.61	−2.99	3.96	8.68	3.57
Rt superficial masseter	−0.9	−0.02	−0.88	−3.39	−0.02	−3.37	3.77	1.21	3.83
Rt deep masseter	−0.69	−0.3	−0.39	−2.75	−0.43	−2.32	3.99	1.43	5.96
Rt medial pterygoid	−0.41	0.01	−0.42	−1.38	0.14	−1.52	3.37	13.84	3.62
Rt condyle	3.62	0.00	3.62	13.08	0.00	13.08	3.61	n/a	3.61
Lt P_3_–M_1_ bite force	−0.55	0.21	−0.76	0.08	0.91	−0.84	−0.14	4.36	1.10
summed torques	−1.43	−0.14	−1.29	−3.29	−0.10	−3.19	2.30	0.68	2.47

### 3.3. Deformation and strain regimes: working (chewing) side corpus

A comprehensive view of the normal (electronic supplementary material, figure S4) and shear (electronic supplementary material, figure S5) strains in the human working side corpus shows that, as hypothesized, the predominant deformation regimes are anterior–posterior twisting and sagittal bending. This corroborates the moments analysis, which indicates that the highest moments experienced on the working side are moments about Y (AP twisting) and Z (sagittal bending) axes (figure 3).

**Figure 4.**
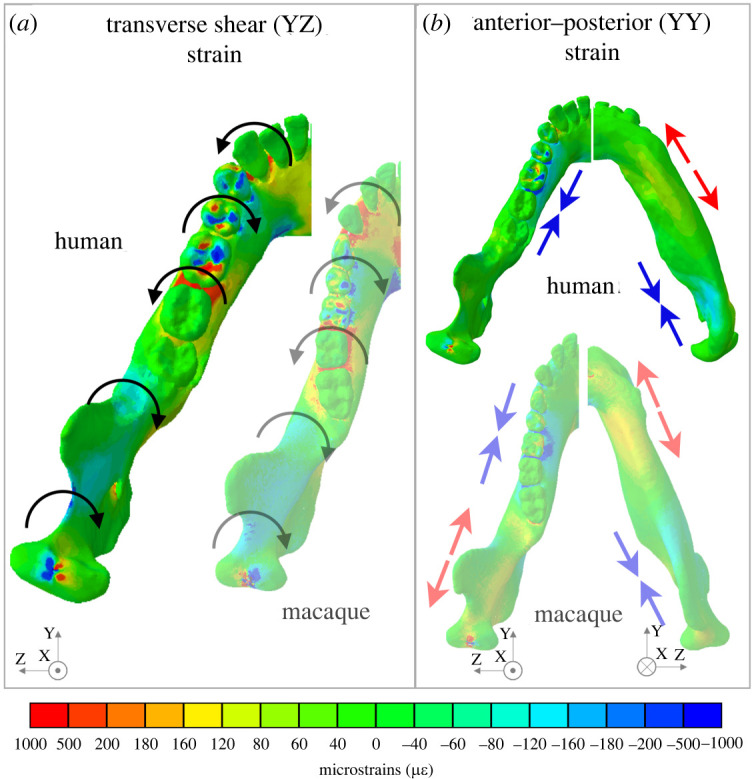
Strain regimes in the working side of the human and macaque mandible during unilateral post-canine chewing described using a common coordinate system: with the origin at the midpoint between the condyles, the *x*-axis in the superior inferior direction, the *y*-axis in the anterior posterior direction—positive anterior, and the *z*-axis in the mediolateral direction—positive toward the left. (*a*) Anterior–posterior twisting, denoted by curved arrows, is associated with specific patterns of positive and negative transverse (YZ) shear strain. Per mandibular coordinate system notation (defined in [17]), working side positive YZ shear strain corresponds to toothrow eversion, i.e. negative AP twisting. Working side negative YZ shear strain corresponds to toothrow inversion, i.e. positive AP twisting. (*b*) Negative sagittal bending is associated with specific patterns of positive and negative anterior posterior (YY) normal strains. For strain regimes, warm colours indicate positive strain and cold colours indicate negative strain. Figures not to scale. Macaque figures adapted from previously published data [17].

The working side corpus undergoes negative sagittal bending posterior to M_1_ and positive sagittal bending at the bite point (electronic supplementary material, figure 5*o*) and is associated with positive basal AP strains underneath the bite point and negative basal AP strains at the mandibular angle (figure 4; electronic supplementary material, figure S4*k*). Corresponding alveolar AP strains are only negative at the bite point and are low elsewhere (figure 4; electronic supplementary material, figure S4*l*). This sagittal bending is accompanied by alternating twisting regimes around the anterior–posterior axis (figure 4*a*). Negative transverse shear strains are observed along the medial planum triangulare, the endocondylar ridge and the recessus mandibulae (figure 4*a*). This gives way to positive transverse shear at the medial pterygoid fossa (electronic supplementary material, figure S5*b*) and the medial prominence beneath M_1_, followed by negative transverse shear strains at the planum alveolare beneath P_1_ and P_2_ (figure 4*a*). The basal pattern of transverse shear strains (electronic supplementary material, figure S5*e*) reflects the AP twisting moments suggesting negative and positive twisting anterior and posterior to the coronoid tip respectively. The complex pattern of transverse shear strain on the planum alveolare indicates additional twisting regimes: negative transverse shear at the planum alveolare of P_3_–P_4_ and anterior segment of M_1_, indicating positive AP twisting. However, as these strain patterns are not reflected on the basal surface (as would be the case if the beam was being twisted), they are probably a direct result of bite point reaction forces. Anterior–posterior (YY) strains on the lingual and buccal surfaces of the corpus and ramus of both the working side were low (electronic supplementary material, figure S4*h,i*), corroborating low transverse bending moments (figure 3). However, no distinct pattern of negative to positive AP normal strains is visible (electronic supplementary material, figure S4*h*,*i*), as expected given the low magnitudes of the corresponding positive and negative SI moments (figure 3).

### 3.4. Deformation and strain: balancing (non-chewing) side corpus

**Figure 5.**
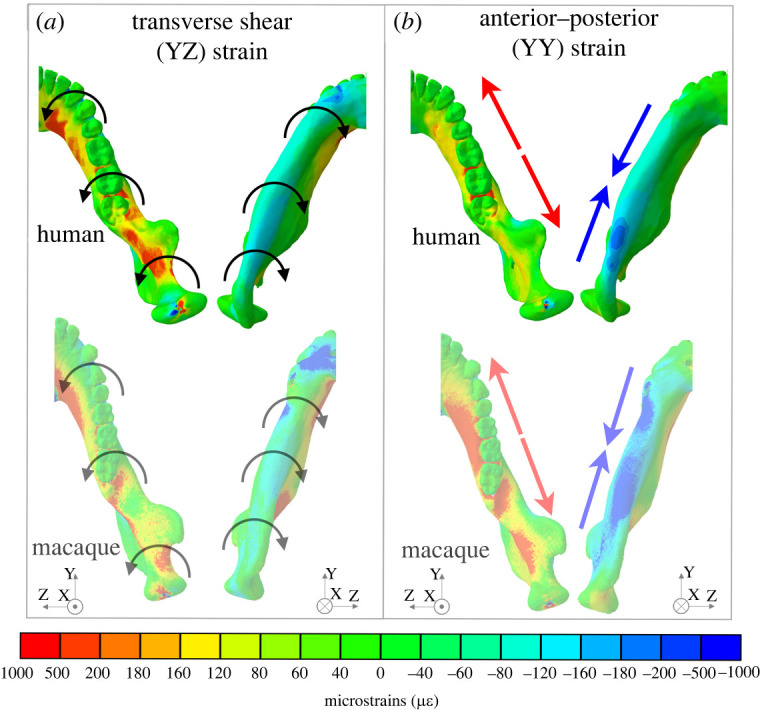
Strain regimes in the balancing side of the human and macaque mandible during unilateral post-canine chewing described using a common coordinate system: with the origin at the midpoint between the condyles, the *x*-axis in the superior inferior direction, the *y*-axis in the anterior–posterior direction—positive anterior, and the *z*-axis in the mediolateral direction—positive toward the left. (*a*) Positive transverse (YZ) shear strain along the toothrow is associated with AP twisting and toothrow inversion, notated per the coordinate system as negative AP twisting, and depicted as curved arrows. (*b*) Positive and negative anterior posterior (YY) normal strains associated primarily with sagittal bending—indicated by red and blue arrows. For strain regimes, warm colours indicate positive strain and cold colours indicate negative strain. Figures not to scale. Macaque figures adapted from previously published data [17].

As predicted, normal and shear strain regimes (figure 5; electronic supplementary material, figures S4 and S5) show that during unilateral post-canine chewing the balancing side corpus of the human mandible predominately undergoes negative AP twisting (toothrow inverted, inferior border everted), negative sagittal bending (convex superiorly) and lateral transverse bending. Negative AP twisting is associated with positive transverse shear strains on the lingual corpus at the planum alveolare and negative transverse shear along the inferior border of the corpus (figure 5*a*; electronic supplementary material, figure S5*e–f*). Negative sagittal bending is associated with positive (tensile) AP strains in the medial planum triangulare, pharyngeal crest, external oblique line and the interalveolar septa, alveolar processes and planum alveolare from M_3_ to P_1_ (figure 5*b*; electronic supplementary material, figure S4*k*,*l*). Negative (compressive) AP strains along the inferior border of the corpus peak at the mandibular angle (electronic supplementary material, figure S4*n*; figure 4*b*) and positive (tensile) AP strains along the superior border peak at the alveolar bone between M_2_ and M_3_. Lateral transverse bending is associated with positive (tensile) AP strains at the planum alveolare of the anterior dentition and negative AP strains at the lateral planum triangulare and the lingual surface of the anterior corpus (electronic supplementary material, figure S4*h*,*j*). These interpretations of strain patterns are expected given the negative SI (lateral transverse bending), negative AP (twisting) and positive ML (sagittal bending) moments (figure 3).

### 3.5. Deformation and strain: symphysis

**Figure 6.**
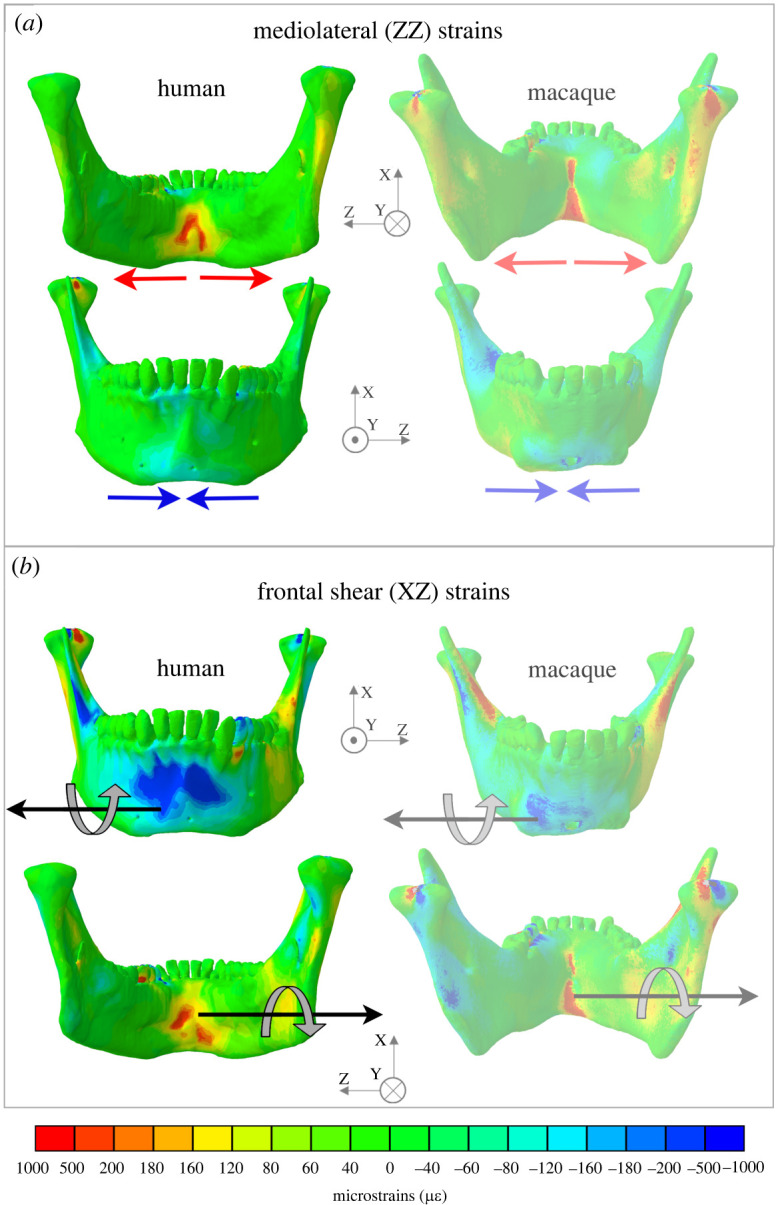
Symphyseal strain regimes in the human and macaque mandible during unilateral post-canine chewing using a common coordinate system: the origin at the midpoint between the condyles, the *x*-axis in the superior inferior direction, the *y*-axis in the anterior–posterior direction—positive anterior, and the *z*-axis in the mediolateral direction—positive toward the left. (*a*) Lateral transverse bending is associated with negative labial and positive lingual mediolateral (YY) strains. (*b*) Negative mediolateral twisting (indicated by light grey arrow) is associated with negative frontal shear labially and positive frontal shear lingually. Figures not to scale. Macaque figures adapted from previously published data [17].

In the symphysis, negative SI (transverse bending) moments (figure 3) result in lateral transverse bending, which is associated with negative ML (ZZ) strains on the labial surface and positive ML strains on the lingual surface (figure 6*a*; electronic supplementary material, figure S4*a*,*b*). Negative ML strains in the labial symphysis are less than 150 µ*ε* and positive ML strains in the lingual symphysis are over 150 µ*ε*, as in macaques and as expected on the basis of curved beam theory [21]. In addition, negative ML (twisting) moments (figure 3) are associated with negative frontal shear strains on the labial surface and positive frontal (XZ) shear strains on the lingual surface (figure 6*b*; electronic supplementary material, figure S5*g*,*h*). In humans, negative frontal shear strains in the labial symphysis extended laterally from the mental protuberance, filling the floor of the mental fossae from the alveoli to the superior border of the mental tubercles. Positive frontal shear strains on the lingual surface surround the genial tubercles and extend along the medial prominence to P_3_ bilaterally (figure 6*b*). The smaller inferior transverse torus of humans is associated with lower ML axial strains and lower frontal shear strains in this region of the symphysis.

### 3.6. Post-fracture fixation

#### 3.6.1. Moments

**Figure 7.**
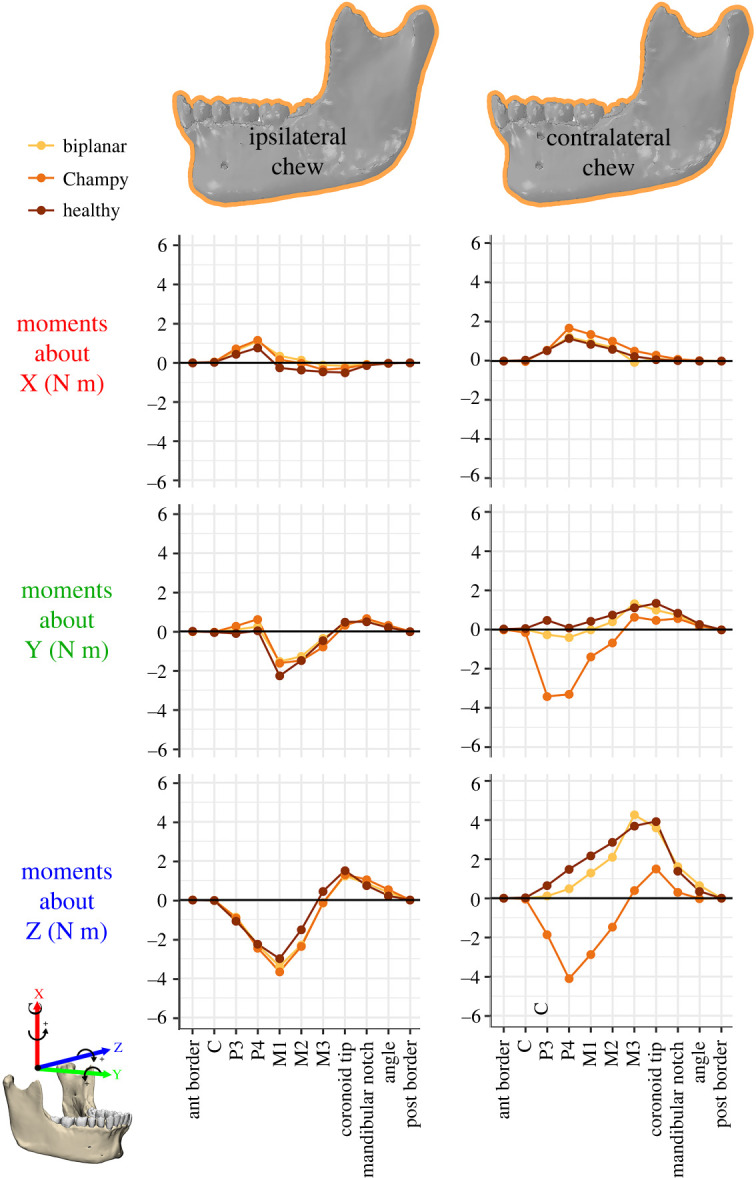
Moments (N m) acting anterior to coronal sections through the left side human mandible models post fracture and fixation during simulation of ipsilateral (left) and contralateral (right) chewing. *Macaca* moments are scaled isometrically to the mandible length of the *Homo* model (length^3^). Note: macaque figures were adapted from previously published data [17]. Moments acting on the right mandible are in electronic supplementary material, figure S6.

Mediolateral (Z), superior–inferior (X) and anterior–posterior (Y) moments acting on the human jaw anterior to coronal cross-sections through the left side mandible during an ipsilateral chew are similar in the healthy and fixation models (figure 7). In both human fixation models, SI moments (about X) anterior to the fracture point are within 0.5 N m of the healthy control (figure 7). AP moments (about Y) are similar between healthy and fixation models, with differences less than 1.0 N m at the bite point (P_3_, P_4_, M_1_) (figure 7). ML moments (about Z) are slightly larger (less than 1.0 N m) in fixation models posterior to P_4_. Champy fixation results in a slightly greater increase in ML moments than biplanar fixation, though the difference was less than 0.5 N m (figure 7). Thus, even though Champy fixation concentrated the load path through a single fixation plate, it does not substantially alter the bending and twisting moments acting on the left side corpus during ipsilateral chews (figure 7).

However, fixation has a larger effect on bending and twisting moments during contralateral chews (figure 7). The largest differences between the healthy control and fixation models are in the magnitude and direction of ML and AP moments (figure 7). Both fixation methods reverse the direction of AP moments (about Y) between P_3_ and M_2_, which are positive in the healthy control but negative in the fixation models. However, while biplanar fixation is within less than 1.0 N m of the healthy control, AP moments in Champy fixation between P_3_ and M_2_ differ by greater than 3 N m from the healthy control (figure 7). Finally, Champy fixation reverses the direction of ML moments anterior to the M_3_ and the moments differ by greater than 5 N m from the healthy control, such that there is negative sagittal bending of the corpus (tension inferiorly and compression superiorly) (figure 7). ML moments anterior to the M_3_ are within less than 1 N m of the healthy control during contralateral chewing under biplanar fixation. Thus, during contralateral chews, Champy fixation substantially alters the loading regime of the jaw.

### 3.7. Bone-implant interface strains

**Figure 8.**
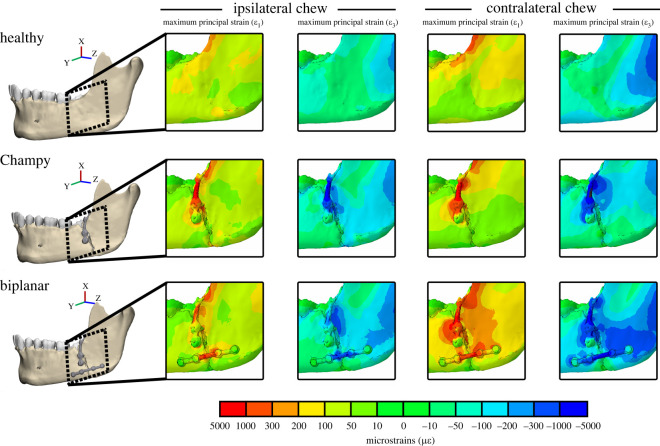
Maximum (*ε*_1_ indicative of tension) and minimum (*ε*_3_ indicative of compression) principal strains in the bone implant interface of the biplanar and Champy angle fracture fixation treatments and in the healthy control during ipsilateral and contralateral chews of the human mandible. Scale bar indicates strain magnitudes in microstrain. Warm colours: positive strains. Cold colours: negative strains.

In humans, for both biplanar and Champy fixation, maximum (*ε*_1_) and minimum (*ε*_3_) principal strain of the cortical bone surrounding the bone-implant interface are greater in magnitude during contralateral chewing and biplanar fixation (figure 8). For both contralateral and ipsilateral chewing, biplanar fixation results in larger principal strains than Champy fixation, with an increase in average magnitude of *ε*_1_ of 96% and 21% respectively, and an increase in average magnitude of *ε*_3_ of 69% and 14% (electronic supplementary material, figure S7). Contralateral chewing, relative to ipsilateral chewing, also increases principal strains, with an increase in average magnitude of *ε*_1_ of 72% and 6% for biplanar fixation and Champy fixation respectively, and a change in magnitude of *ε*_3_ of +38% and −7% (electronic supplementary material, figure S7).

Under Champy fixation, strains are concentrated along the external oblique line and the superior half of the fracture plane (figure 8). In biplanar fixation, additional areas of high strain border the inferior fracture plane, as well as posterior to the fracture plane toward the masseteric fossa (figure 8). These concentrated areas of strain drive the observed increases in average cortical principal strain during contralateral chewing (figure 8). During contralateral chewing, under biplanar fixation, in the region surrounding the bone-implant interface, there is a 2.8×, 2.7× and 11.9× increase in the number of cortical bone surface nodes with maximum principal strain (*ε*_1_) values of 200–300 µ*ε*, 300–1000 µ*ε* and 1000–5000 µ*ε* respectively (electronic supplementary material, figure S7). Under Champy fixation, the number of cortical nodes in the region surrounding the bone-implant interface with *ε*_1_ values in those ranges increase by 1.5×, 1.7× and 15.1×, respectively (electronic supplementary material, figure S7). Minimum principal strains are similarly larger in magnitude during contralateral chewing and follow a pattern of distribution similar to maximum principal strains (electronic supplementary material, figure S7).

### 3.8. Interfragmentary strain

**Figure 9.**
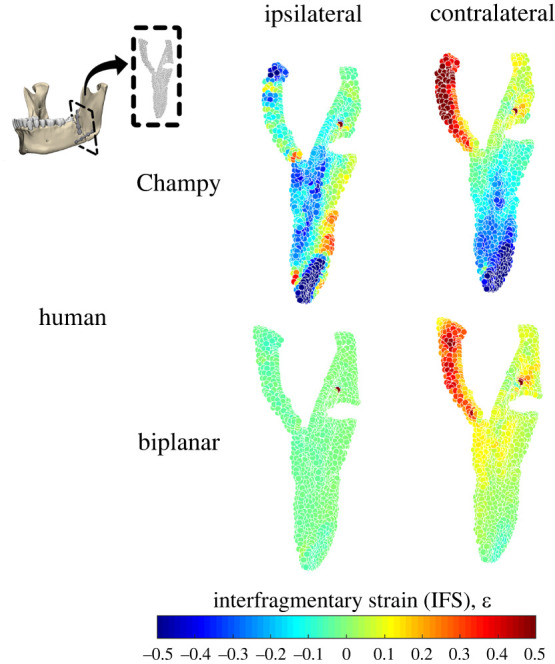
Interfragmentary strain (IFS) between nodes across the fracture plane during chewing ipsilateral and contralateral to the fracture in humans and macaques. Warm and cold colours show areas with positive (gap widening) and negative (gap shortening) IFS respectively.

In humans, the least interfragmentary strain occurs under biplanar fixation (table 4 and figure 9), with an average magnitude of 0.013 and 0.065 *ε* interfragmentary strain (IFS) during ipsilateral and contralateral chewing respectively (table 4). In biplanar fixation, the increased IFS during contralateral chewing is driven by increased displacement in the tensile regions (+0.003 versus +0.070 *ε*, table 4), predominantly on the lingual side of the superior fracture plane (figure 9). Larger magnitudes of IFS occur under Champy fixation, with 0.114 and 0.127 *ε* during ipsilateral and contralateral chewing respectively (table 4). Similar to biplanar fixation, the increased magnitude of IFS in Champy fixation during contralateral chewing is driven by increased IFS in the tensile regions, predominantly on the lingual side of the superior fracture plane (figure 9). However, there are also significant regions of compression in both contralateral and ipsilateral chewing that are not present under biplanar fixation. This included regions with −0.4 to −0.5 *ε* of IFS at the inferior buccal border during both ipsilateral and contralateral chewing, and additional regions with −0.1 to −0.3 *ε* of IFS along the lingual aspect during ipsilateral chewing (table 4 and figure 9).

**Table 4.** Average (median) interfragmentary strain (*ε*) for different mandibular angle fracture fixation finite-element models under chewing loads.

		all regions, *ε* (avg. absolute value)	compressive regions, *ε*	tensile regions, *ε*
ipsilateral chew	Champy	0.114	−0.137	+0.056
	biplanar	0.013	−0.015	+0.003
contralateral chew	Champy	0.127	−0.136	+0.095
	biplanar	0.065	−0.029	+0.070

### 3.9. Global bone strain regimes post-fracture fixation

**Figure 10.**
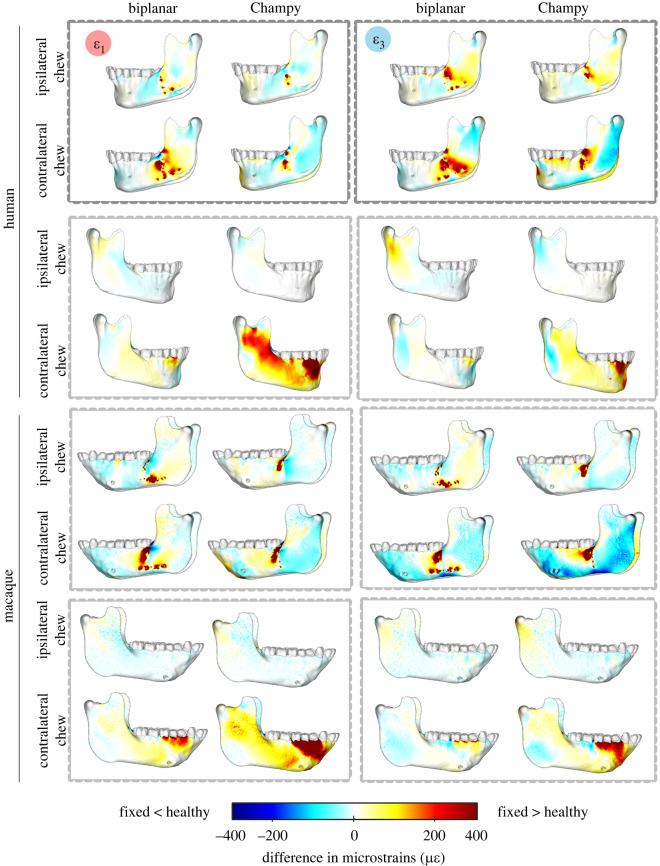
Differences in maximum (*ε*_1_) and minimum (*ε*_3_) principal strain magnitudes between healthy control and fracture models repaired with Champy or biplanar technique; simulations of chews ipsilateral and contralateral to the fracture in the left side mandible. N.B.: ipsilateral refers to a bite point on the left side; contralateral refers to a bite point on the right side. Scale bar: difference in microstrain between fixed and healthy models in both humans and macaques, for *ε*_3_ principal strain difference is calculated as differences of absolute values. White: no difference in strain magnitudes. Warm colours: larger strains in fixed than control model. Cold colours: lower strains in fixed than control model.

In ipsilateral chewing, biplanar and Champy fixation result in similar deviations in bone strain regimes from the healthy control. Under both biplanar and Champy fixation, on the fractured side of the mandible maximum principal strains (*ε*_1_) are 50–100 µ*ε* lower than the healthy control at the lateral prominence of the corpus. On the unfractured side of the mandible they are within 0–50 µ*ε* of the healthy control along the corpus, angle, ramus and condyle. Under Champy fixation the condyle and external oblique line on the unfractured side show lower and higher *ε*_1_ than the healthy control respectively, with the opposite pattern observed in biplanar fixation (figure 10). Under both fixations, minimum principal strains (*ε*_3_) on the fractured side of the mandible are 50–100 µ*ε* higher at the masseteric fossa and 0–50 µ*ε* lower at the lateral prominence of the corpus (figure 10). Proximal to the fracture plane, on the superior aspect, minimum principal strains are 200–400 µ*ε* higher than the healthy control in both biplanar and Champy fixation (figure 10). On the unfractured side, at the condylar neck, *ε*_3_ is 50–150 µ*ε* higher in biplanar fixation and 50–100 µ*ε* lower in Champy fixation (figure 10).

Excluding the area proximal to the fracture plane, contralateral chewing results in larger deviations from the healthy control in the global bone strain regime than ipsilateral chewing. This difference is most prominent on the unfractured side of the mandible under Champy fixation, which shows 200–400 µ*ε* increases in *ε*_1_ at the condylar neck, the sigmoid notch, the crista ectacondyloidea, the external oblique line and the alveolar process in the parasymphysis (figure 10). Under biplanar fixation, all of these regions are within 0–50 µ*ε* of the healthy control. Similar trends, albeit at lower magnitudes, were observed for *ε*_3_ (figure 10). On the fractured side of the mandible, relative to the healthy control, biplanar fixation results in 100–400 µ*ε* increases in *ε*_1_ and *ε*_3_ proximal to the fracture plane and at the masseteric fossa of the angle (figure 10). Champy fixation also results in 100–400 µ*ε* increases in *ε*_1_ and *ε*_3_ proximal to the fracture plane but these changes are limited to the superior border of the fracture and along the external oblique line (figure 10). In addition, Champy fixation results in 50–100 µ*ε* lower *ε*_1_ and *ε*_3_ than the healthy control from the condylar process along the masseteric tuberosity to the posterior angle, and at the inferior border of the corpus (figure 10). Biplanar fixation results in 50–100 µ*ε* lower *ε*_1_ and *ε*_3_ along the condylar process but strains along the basal aspect of the corpus are within 0–50 µ*ε* of the healthy control (figure 10).

## 4. Discussion

Our results confirm that deformation and accompanying strain regimes are similar in healthy adult human and macaque mandibles (figures 3–6), and that the effects of fixation are similar between species.

In both species the healthy balancing side corpus undergoes lateral transverse bending, negative AP twisting (inversion of toothrow) and negative sagittal bending; the working side undergoes negative AP twisting (eversion of toothrow), positive sagittal bending about the bite point and negative sagittal bending in the ramus; and the symphysis undergoes lateral transverse bending and negative mediolateral twisting (figures 3–6). The most marked differences between species are in patterns of SI (transverse bending) moments. Humans experience lower transverse bending moments at the symphysis and at working and balancing side corpora, probably due to the less ML-oriented jaw muscles (table 2) (figure 3). On the balancing side, AP twisting moments acting in the molar region are greater (more negative, greater inversion of toothrow) in humans than in macaques (figure 3). The relatively greater (negative, toothrow eversion) AP moments acting on the working side corpus at M_1_ are not likely to be due to the more medial position of the human M_1_ relative to AP axis, as this would decrease the twisting moment of a vertical component of bite force. It is more likely to be due to the greater medial component of medial pterygoid muscle force acting below the twisting axes, acting to evert the toothrow. As noted, this more medially oriented medial pterygoid is associated with the increased inter-rami distance in humans (figure 2).

Differences in strain regimes between species are greatest at the mandibular symphysis, where the frontal shear strains associated with ML twisting are concentrated superiorly and laterally to the mental protuberance in the human models, versus inferiorly at the symphysis in the macaque models. The two models show only minor differences in ML twisting moments acting on the symphysis, suggesting the differences in strain regime are due to differences in symphyseal morphology [16,26,28]. Similarity in deformation and strain regimes in human and macaque mandibles are in concert with the suggestions of others [47–49], that ‘*masticatory mechanics are highly conserved among anthropoids*' ([49], p. 323).

The effects of fixation are also consistent between humans and macaques. In humans, Champy fixation results in more interfragmentary displacement than does biplanar fixation, especially during contralateral chewing (figure 9 and table 4), which corresponds to previous macaque results [15]. In both macaques and humans, both Champy and biplanar fixation essentially recover the moments acting on a healthy mandible during chewing ipsilateral to the fracture. During chewing contralateral to the fracture, biplanar fixation better recovers the moments observed in the healthy mandible, whereas Champy fixation is associated with significant deviations from the healthy condition (figure 7). During contralateral chewing, Champy fixation is associated with a switch from inversion to eversion of the balancing side (fractured) corpus, and a switch from positive to negative sagittal bending moments. Both models show very similar patterns of differences between healthy and fracture-fixed strain regimes on the right side during chewing ipsilateral and contralateral to the fracture (figure 10). When chewing on the same side as the fracture (left), the strain regime on the right side of both macaques and humans shows minimal divergence from the strain regime in the healthy model. Similarly, when chewing contralateral to the fracture (right), the right side mandible shows elevated *ε*_1_ magnitudes in the parasymphyseal region, below and anterior to the bite point, and in a strip running across the ramus toward the neck of the mandible; in both species this effect is greater after Champy repair than biplanar. Contralateral (right side) chewing following Champy repair is also associated with increased *ε*_3_ magnitudes in the same areas in both species, suggestive of an increased shear regime, probably associated with increased twisting of the working side mandible. In both species, there are only small changes to *ε*_3_ magnitudes on the right side during right-side chewing following biplanar repair of a left fracture (figure 10).

No published studies of fracture repair in humans provide suitable comparisons for our fracture-fixed FE models. Previous comparisons of angle fracture fixation techniques have used benchtop experiments with cadaveric or resin-based human mandibles [50–52]. These have yielded conflicting results, probably because recreating the physiological loading environment of the mandible using benchtop force testers, pulleys or weight constructs is very difficult [53]. Published studies of angle fracture fixation using FE models corroborate aspects of our results, but variations in methodology preclude the use of any one model as a benchmark for comparison. For example, Xu *et al*. [54], Wang *et al*. [55] and Arbag *et al*. [56] all show increased interfragmentary displacement for single-plate fixation relative to biplanar fixation. Further corroborating our results, Wang *et al.* [55] found patterns of interfragmentary displacement similar to ours during contralateral chewing, with the superior lingual border and inferior aspect of the fracture plane experiencing the highest magnitudes of displacement. However, the aforementioned models used simulated fracture planes with non-physiological muscle activation. The FE models in Liu *et al*. [11], Xu *et al*. [54] and Wang *et al*. [55] were loaded at the bite point, muscles were modelled as springs, and contralateral and ipsilateral molar and incisal loading were modelled by keeping all muscle spring conditions the same while varying the bite point. The FE models in Arbag *et al*. [56] did not apply muscle loads, but rather exclusively used a point load on the incisor to represent biting. This is in contrast to our models, which use working side and balancing side muscle activations representative of physiological chewing, and a fixed boundary condition for the bite points as opposed to a point load. As such, our models probably better approximate the actual loading of the mandible during chewing, and it is not feasible to make a meaningful comparison of the measured strains and stresses between our models and prior studies.

Given the limitations to *in vivo* human experiments (ethical and practical), mandibular and maxillofacial research has relied heavily on animal models, particularly sheep [52,57–60]. However, a recent study [61] has highlighted that sheep are unsuitable models for human mandibular biomechanics due to differences in the post-fixation mechanical environment of the jaw between species. Specifically, they found that during bilateral intercuspal (all post-canine dentition) and incisal clenching, and unilateral (M_1_, P_4_) clenching, human FE models exhibit lower peak von Mises stresses in their plate constructs (difference approx. 50 MPa) and higher global principal strains (maximum and minimum—difference approx. 150 µ*ε*) than sheep FE models. Orassi *et al*. [61] did not provide an in-depth analysis of the deformation and strain regimes in the sheep jaw, so it is difficult to ascertain whether these dissimilarities in peak principal bone strain and peak von Mises plate stress are reflective of differences in bending and deformation regimes between humans and sheep. Still, their findings, as well as the findings of this study, suggest that the animal model used for maxillofacial and craniofacial research needs to shift away from the sheep model and move toward animals that chew more similarly to humans, such as macaques. Ultimately, the similarities between the mechanical environment of the human and macaque mandible, both healthy and post-fracture fixation, suggest that macaques make an excellent pre-clinical animal model for research into mandibular biomechanics and fracture repair.

Finally, while the results of this and prior studies [15] support the use of biplanar fixation over Champy fixation in the treatment of mandibular angle fracture, a key limitation to all studies to date, including ours, is a dearth of knowledge on muscle activation patterns in humans and animal models post-fracture. In this study and in our previous work we loaded all fracture-fixed FE models with muscle activations estimated using data from a healthy macaque [17]. However, fracture and fixation may alter muscle activation patterns and jaw kinematics [62,63]. Studies of condylar fracture have shown that the mastication forces are reduced up to a year post-fracture, with changes in jaw kinematics persisting for 2 years [64,65]. Thus, there are probably multiple drivers of changes in mastication following fracture fixation. Acutely, soft-tissue damage secondary to the fracture and iatrogenic damage during the repair probably alter contraction forces and muscle recruitment. This alteration might be particularly apparent in cases of biplanar fixation, as the additional surgical entry point of biplanar fixation may result in more masseteric muscle disruption than Champy fixation, which relies on a single trans-oral entry [9,66]. However, once the soft-tissue damage heals, a different set of drivers, including learned changes in masticatory behaviour, damage to reflex pathways and reactionary changes to abnormal strain/stress in the bone and soft tissue probably drive the continued variations in mastication. Of note, these deviations in the kinematics of mastication have been observed to be largest during contralateral chewing [65], and there is evidence to suggest a preferential tendency to chew contralateral to the side of mandible fractures [67]. As such, a deeper understanding of mastication in a post-fracture environment is necessary for more accurate FE models. In addition, it is important to highlight that even the most accurate FE models cannot completely predict clinical outcomes. Clinical studies reporting favourable outcomes for one fixation method over another [12,68] could be driven by factors not accounted for by an FE model, such as surgical ease of operation, surgeon experience with the procedure, and iatrogenic damage secondary to the fixation.

Mandibular fractures remain, despite their prevalence, surgical problems with no perfect solution. However, there is a path to improved patient outcomes with fewer complications, and it begins with studying the appropriate pre-clinical animal models to develop our understanding of mechanics and mastication in the post-fracture fixation environment. Armed with models that accurately reflect the mechanics of a fractured human model, we can more appropriately characterize the mechanical landscape under differing fixation approaches and chewing behaviours. Using that knowledge, we will ultimately be able to tailor fixation approaches and physical rehabilitation to mirror the physiologic healthy condition, thereby improving patient outcomes. Our results provide key foundational pieces of knowledge along this path: first that the congruity in mechanical environments between macaques and humans makes macaques an excellent pre-clinical option, and second, that Champy fixation may result in unfavourable mechanics relative to biplanar fixation, especially during contralateral chewing.

## Data Availability

All finite-element models and data analysis can be found on Monash Figshare at https://figshare.com/s/ee9a709b7f288f45e8b6 and https://figshare.com/s/c0b4203132efbd800ec6 respectively. Data analysis files involve raw data, relevant code (R, Python and Matlab) and output data or figure files. The data are provided in electronic supplementary material [69].
